# Mechanistic Insight into Orthodontic Tooth Movement Based on Animal Studies: A Critical Review

**DOI:** 10.3390/jcm10081733

**Published:** 2021-04-16

**Authors:** Hyeran Helen Jeon, Hellen Teixeira, Andrew Tsai

**Affiliations:** Department of Orthodontics, School of Dental Medicine, University of Pennsylvania, 240 South 40th Street, Philadelphia, PA 19104-6030, USA; hellen@dental.upenn.edu (H.T.); andrewts@dental.upenn.edu (A.T.)

**Keywords:** orthodontic tooth movement, animal studies, mechanosensing, osteoclastogenesis, osteogenesis

## Abstract

Alveolar bone remodeling in orthodontic tooth movement (OTM) is a highly regulated process that coordinates bone resorption by osteoclasts and new bone formation by osteoblasts. Mechanisms involved in OTM include mechano-sensing, sterile inflammation-mediated osteoclastogenesis on the compression side and tensile force-induced osteogenesis on the tension side. Several intracellular signaling pathways and mechanosensors including the cilia and ion channels transduce mechanical force into biochemical signals that stimulate formation of osteoclasts or osteoblasts. To date, many studies were performed in vitro or using human gingival crevicular fluid samples. Thus, the use of transgenic animals is very helpful in examining a cause and effect relationship. Key cell types that participate in mediating the response to OTM include periodontal ligament fibroblasts, mesenchymal stem cells, osteoblasts, osteocytes, and osteoclasts. Intercellular signals that stimulate cellular processes needed for orthodontic tooth movement include receptor activator of nuclear factor-κB ligand (RANKL), tumor necrosis factor-α (TNF-α), dickkopf Wnt signaling pathway inhibitor 1 (DKK1), sclerostin, *transforming growth factor* beta (*TGF-*β**), and bone morphogenetic proteins (BMPs). In this review, we critically summarize the current OTM studies using transgenic animal models in order to provide mechanistic insight into the cellular events and the molecular regulation of OTM.

## 1. Introduction

Alveolar bone remodeling in orthodontic tooth movement (OTM) requires the coordinated action of different cell types, including periodontal ligament (PDL) fibroblasts, mesenchymal stem cells, inflammatory cells, osteoblasts, osteocytes, and osteoclasts. Generally, OTM is composed of three stages on the compression side; (i) a gradual compression of the PDL, which may last from about 4–7 days, (ii) the hyalinization period, when cell death due to lack of blood supply in the compressed area of the PDL occurs, which may last from 7–14 days or more, and (iii) the secondary period, which is characterized by direct bone resorption so that the tooth will continue to move [1,2,3]. On the tension side, the PDL is stretched and blood flow is activated, stimulating osteoblastic activity and osteoid deposition and mineralization. On mechanical force loading, cells around the tooth sense either compression or tension and release multiple cytokines and growth factors that stimulate subsequent biological responses. The process by which cells transmit mechanical forces and generate biological responses is essential for bone remodeling in OTM [4].

On the compression side, multinucleated osteoclasts initiate bone resorption to allow tooth movement to occur in the direction of the applied force, which is a rate limiting step in OTM. In addition, a sterile inflammatory response is induced by the generation of proinflammatory cytokines such as tissue necrosis factor (TNF), interleukin-1 (IL-1), prostaglandins, and IL-6, along with matrix metalloproteinases (MMPs) within a short time after the application of pressure [5,6,7,8,9,10]. The response to mechanical stress induces transitory inflammation that is pathogen-free. In addition, prostaglandins are secreted when cells are mechanically deformed and focal adhesion kinase, the mechanosensor in PDL cells, is known to be related with this process [11]. Therefore, both sterile inflammation and mechano-transduction are important for OTM [12]. Cells experiencing compressive forces induce osteoclastogenesis through up-regulation of receptor activator of nuclear factor kappa-Β ligand (RANKL) [13]. Proinflammatory cytokines induce RANKL expression to stimulate osteoclastogenesis, further contributing to bone resorption in OTM [14]. On the tension side, PDL cells are stretched and proliferate with increased PDL width, followed by new bone formation, eventually returning to a normal PDL width [15]. Progenitor cells in the PDL and alveolar bone proliferate and differentiate into osteoblasts to produce new bone. The osteogenic transcription factor Runx2 and bone matrix proteins osteocalcin and osteopontin are significantly up-regulated by tension forces [7]. The mechano-response, osteoclastogenesis and osteogenesis are important components of OTM as they represent simultaneous bone remodeling processes in response to mechanical loading.

Human studies examining changes induced by orthodontic forces have frequently examined gingival crevicular fluid after orthodontic force loading. While important, these studies do not establish the cause and effect relationships. Transgenic mouse models are ideal in delineating the molecular actions of specific genes, as they facilitate lineage-specific gene deletion to well-defined cell types [16]. In addition, inducible transgenic mice models are available, allowing the induction of a transgene or the deletion of an endogenous gene in a time- and tissue-specific manner to address limitations of global constitutive germ-line deletion [17].

In this review, we focus on the roles of various mechanosensory cells, cytokine expression and signaling pathways in OTM that have been identified by animal models and summarize the possible cellular mechanosensors. A better understanding of the cellular processes in OTM may one day benefit our patients by expediting tooth movement, preventing relapse and improving treatment stability through the modification of specific genes which are critical for the orthodontic bone remodeling.

## 2. Cytokines, Mechanosensory Cells, and Intracellular Signaling Pathways in OTM

### 2.1. Cytokines in OTM

#### 2.1.1. RANKL

RANKL is a member of the TNF cytokine family and is critical for osteoclastogenesis [18]. During OTM, RANKL is highly expressed in periodontal tissue on the compression and tension side [13,19]. Numerous cell types in OTM have been shown to express RANKL including PDL fibroblasts, mesenchymal stem cells, lymphocytes, osteoblasts, and osteocytes, particularly in response to inflammatory cytokines [9,13,20,21,22].

Human gingival crevicular fluid (GCF) samples have been used for cytokine analysis during OTM as it is non-invasive and convenient. Human GCF isolated from the tooth 24 h after orthodontic forces application, the early phase of OTM, had shown a significant increase in the levels of RANKL, IL-1β, IL-6, and TNF-α, while the levels of osteoprotegerin (OPG) had remained significantly lower when compared to the control teeth [7,23,24,25]. Furthermore, Garlet et al. examined the cytokine expression on the PDL of extracted human teeth after OTM [7]. After 7 days of OTM, teeth were extracted and PDL cells on both compression and tension sides were collected for real-time PCR analysis. On the compression side, tumor necrosis factor alpha (TNF-α), RANKL, and matrix metalloproteinases (MMPs) were highly expressed. On the tension side, IL-10, tissue inhibitors of metalloproteinase 1 *(TIMP-1)*,type I collagen, OPG, and osteocalcin were highly expressed. The same author tested chemokine expression on the extracted teeth after OTM and found that the compression side exhibited higher expression of monocyte chemoattractant protein-1 (MCP-1/CCL2), macrophage inflammatory protein-1α (MIP-1α/CCL3) and RANKL, which predominate bone resorption activity, while the tension side presented higher expression of osteocalcin.

Consistent with human studies, animal models demonstrated bone resorption activity with proinflammatory cytokines and osteoclastic markers on the compression side and bone formation activity with the osteogenic markers on the tension side. To further apply the findings to clinical orthodontics, several animal studies have examined the modulation of RANKL to accelerate OTM [26,27,28]. Injection of RANKL during OTM increases osteoclastogenesis and the rate of tooth movement [26]. Indeed, the rate of OTM is increased by 130% with RANKL injection [28]. Local RANKL gene transfer in animals with OTM increases RANKL protein expression and osteoclastogenesis without any systemic effects, accelerating the amount of tooth movement [27]. The authors proposed that local RANKL gene transfer might be a useful tool to accelerate orthodontic tooth movement, even the ankylosed teeth. Conversely, daily local RANKL antibody injection reduces the rate OTM by 70% [22]. In a comparison of RANKL gene transduction compared to periodontal accelerated osteogenic orthodontics, gene transduction led to more prolonged osteoclastogenesis and a greater rate of tooth movement during OTM [29].

In vitro compressive force causes an increase of RANKL expression and a decrease of OPG expression in human PDL cells, consistent with the human GCF and animal studies results. PDL fibroblasts are distorted under compressive force and express higher amounts of RANKL, TNF-α, MMPs, IL-1β and prostaglandins on the compression side [30]. Experimental compressive forces on the PDL resulted in a 16.7-fold increase in RANKL secretion and a 2.9-fold decrease in OPG secretion when compared to the control [23].

Taken together, studies with RANKL indicate that this cytokine is a central pro-osteoclastogenic factor that is expressed in response to mechanical forces. Interestingly, RANKL is also expressed on the tension side [19]. Thus, early induction of RANKL and osteoclastogenesis in response to tension may initiate a formation of bone remodeling that leads to increased bone formation on the tension side. This concept warrants further investigation.

#### 2.1.2. Sclerostin

Sclerostin, encoded by the SOST gene and is primarily produced by mature osteocytes in response to OTM, promotes bone resorption and inhibits new bone formation [31,32]. Sclerostin stimulates RANKL expression by osteocytes, negatively regulates expression of BMP proteins and prevents canonical Wnt signaling [33,34]. Sclerostin expression is initially induced on the compression side in OTM models and gradually diminishes after 5–7 days, demonstrating their effect in the early phase of OTM [31,35]. During OTM, sclerostin KO mice have a 20% reduction in osteoclasts and reduced RANKL expression on the compression side with a reduced rate of tooth movement [35]. Local injection of sclerostin on the compression side doubles RANKL expression, reduces OPG expression by 30%, increases osteoclastogenesis by 150% and accelerates tooth movement [36]. In addition, in vitro studies showed that rhSCL-supplement enhanced the expression of RANKL and the RANKL/OPG ratio in osteocytes, supporting the in vivo finding. In addition, the intensity of sclerostin expression is closely related with the force magnitude [37]. On the tension side, sclerostin expression is immediately decreased and maintained at low levels during OTM, negating their negative effect on new bone formation [31]. These studies suggest that sclerostin can be a key factor in OTM by regulating both bone resorption and formation.

#### 2.1.3. Bone Morphogenetic Proteins (BMPs)

It is well known that BMPs induce new bone formation and that the expression of BMPs increases on the tension side during OTM, stimulating the differentiation of mesenchymal stem cells to osteoblasts [30,38]. Noggin, an inhibitor of several bone morphogenetic proteins (BMPs), prevents mechanical force-induced osteoblast differentiation. BMP-3 expression is gradually increased on the tension side until 14 days in rodent models of OTM, the mid-stage in OTM [39]. One study examined the effect of BMP2 injection on tension side and found that local injection of BMP-2 on the tension side did not accelerate OTM, indicating that new bone formation per se is not a rate limiting step in OTM [40].

#### 2.1.4. Transforming Growth Factor (TGF)-β

TGF-β signaling is involved in many cellular processes, including cell migration, proliferation, differentiation, and cellular homeostasis [41]. A previous study with extracted human teeth after OTM showed that TGF-β expression was similarly increased in both the compression and tension sides [7]. In OTM, its role on the compression side is complex as TGF-β has both positive and negative effects on osteoclastogenesis [42]. In some studies, TGF-β has been reported to inhibit osteoclastic activity. However, other studies found that TGF-β actually induces bone resorption, depending on the cell types involved, TGF-β concentration, and inducing mechanism [42,43]. Its expression on the tension side is significantly greater than that on the compression side [7,44,45,46]. TGF- β is generally known for its anabolic activity, regulating osteoblast differentiation from progenitors on the tension side [47]. Pretreatment with a TGF-β receptor inhibitor inhibits mechanical force-induced bone mineralization in vitro, suggesting that TGF-β could play a role in osteogenesis in response to tension forces during OTM.

Combining all, the findings from the animal studies could be the base foundation for the studies to expedite the OTM in humans. For example, RANKL or sclerostin can be given on the compression side to accelerate the osteoclastogenesis or BMPs can be given on the tension side to support the new bone formation. As previously mentioned, the new bone formation itself on the tension side cannot make the tooth movement faster while their effects are more important in the late phase in OTM. Therefore, many studies to speed up the velocity of OTM have been focused on the osteoclastogenic markers on the compression side.

### 2.2. The Mechanosensory Cells in OTM

**Figure 1:**
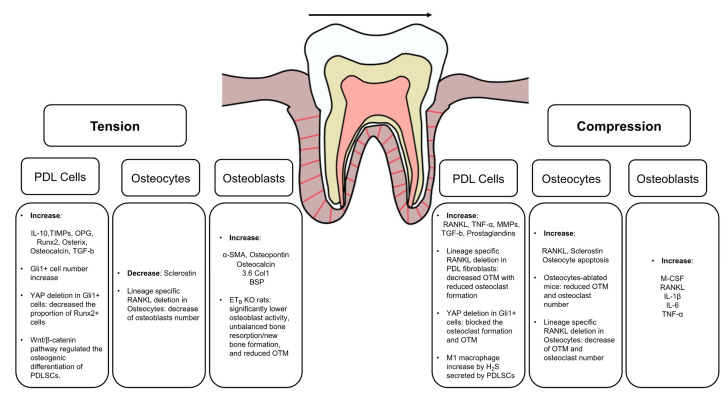
Cytokines and Mechanosensory cells in OTM.

PDL cells, osteocytes, and osteoblasts are the principal mechanosensory cells that produce various cytokines to regulate alveolar bone remodeling in OTM, by converting mechanical force into intracellular signals [48,49,50,51] (Figure 1). The role of mechanosensors in these cells during OTM will be reviewed later in this paper.

#### 2.2.1. Periodontal Ligament Cells

##### Periodontal Ligament Fibroblasts

The periodontal ligament (PDL) is a fibrous tissue that connects teeth with alveolar bone and transmits mechanical stimuli [52]. The PDL comprises of heterogenous cell types including fibroblasts, progenitor cells, bone-lining cells, osteoclasts, endothelial cells, nerve cells and others [53]. PDL fibroblasts constitute 50–60% of the total PDL cellularity and contribute to bone resorption in OTM as a main source of RANKL [21]. Interestingly, PDL fibroblasts have some characteristics similar to those of osteoblasts, expressing a 2.3 kb regulatory unit of Col1α1 promoter typical of osteoblasts and osteocytes [54,55] and bone-associated proteins such as alkaline phosphatase [56]. Indeed, PDL fibroblasts are more similar to tendon cells than skin fibroblasts in many respects [30,57].

On the compression side, the experimental mice with RANKL deletion in PDL fibroblasts showed significantly less osteoclast formation with narrower PDL space compared to control mice, leading to severely impaired OTM [21]. In addition, our recent study found that this up-regulation of RANKL depends on NF-κB activation [58]. NF-κB inhibition in PDL fibroblasts blocked the OTM with significantly reduced osteoclastogenesis, narrower PDL width, higher bone volume fraction and reduced RANKL expression compared to wild type mice. Both studies support the critical role of PDL fibroblasts via NF-κB activation in OTM.

##### Periodontal Ligament Stem Cells

Mesenchymal stem cells reside in the PDL, giving rise to PDL, alveolar bone, and cementum during alveolar bone remodeling. Gli1+ cells have been identified as the multipotent stem cells in adult mouse PDL [59]. Complete removal of Gli1+ cells using the inhibitors or by the genetic ablation significantly reduce OTM by 60% and diminish osteoclast formation by more than 80% [60]. On the tension side, Gli1+ increases its cell number and differentiate into osteoblasts with increased Runx2 expression during OTM [60].

Mesenchymal stem cells reside in the PDL, giving rise to PDL, alveolar bone, and cementum function as mechanosensory cells during OTM [60]. Yes-associated protein (YAP) and the paralogue transcriptional coactivator with PDZ-binding motif (TAZ), the downstream effectors of the Hippo signaling pathway, have been identified as important regulators during mechanotransduction [61]. Recent rodent OTM studies showed that YAP and TAZ expression were up-regulated with nuclear translocation in the PDL cells on both compression and tension side [62,63]. Moreover, YAP and TAZ expression were proportional to the applied orthodontic force. A recent study investigated the role of Gli1+ cells through Yes-associated protein (YAP) activation in mouse OTM models [60].Lineage-specific deletion of the YAP in Gli1+ cells significantly reduced OTM by 50% with decreased osteoclast formation by more than 80% on compression side. On the tension side, the same transgenic mice with the YAP deletion in Gli1+ cells showed a significantly decreased proportion of Runx2+ cells by more than 80%. In vitro cyclic stretch promoted the osteogenic differentiation of human PDL cells [62]. Moreover, the nuclear translocation of YAP was significantly increased with increased expression of connective tissue growth factor (CTGF) and cysteine-rich angiogenic inducer 61 (CYR61) mRNA, the target gene of YAP. Furthermore, knockdown of YAP suppressed the cyclic stretch induced osteogenesis in human PDL cells, while overexpression of YAP enhanced osteogenesis. Both in vivo and in vitro data supportthe role of YAP as the mechanical sensor and important regulator of the osteogenic differentiation in PDL cells under tensile force. In addition, the level of tension is important in the osteogenic differentiation as the magnitude of tension differentially regulates osteogenic and osteoclastic process [64]. Tension with a magnitude of 12% could increase osteogenic differentiation and proliferation of mesenchymal stem cells whereas tension above 12% would up-regulate the function of mesenchymal stem cells to regulate osteoclast differentiation, demonstrating the importance of the light force during OTM especially for the patients with poor bony support such as periodontitis [65,66].

Mechanical force-induced hydrogen sulfide (H_2_S), produced by PDL mesenchymal stem cells, supports macrophage polarization toward an inflammatory, M1 phenotype and promotes osteoclast activity in OTM [67,68]. These cells express cystathionine-β-synthase that generates H_2_S. Treatment with an inhibitor of H_2_S reduces osteoclast formation and OTM by almost half. The generation of M1 macrophages is increased 5.6-fold after orthodontic force loading. An H_2_S blocker reduces M1 macrophage formation by 70%, and an H_2_S donor enhances it 1.4-fold. This shows that PDL mesenchymal stem cells can increase the expression of M1-macrophages, which are main source of several proinflammatory cytokines such as IL-1β, IL-6, and TNF-α, leading to the RANKL stimulation.

#### 2.2.2. Osteocytes

Osteocytes are terminally differentiated from osteoblasts and embedded in the bone matrix. They are the most abundant cells in the adult skeleton, comprising 90–95% of all bone cells [12,51]. They are the primary mechanosensory cells in bone and regulate both osteoclast and osteoblast formation and function during mechanical force-induced bone remodeling [69,70]. They have dendritic processes that interact with other osteocytes and bone-lining cells. Mechanical loading stimulates dentin matrix protein 1 (DMP1) expression in osteocytes *in vivo*, which is a key molecule in regulating osteocyte formation, maturation, phosphate regulation and regulating mineralization [71,72]. In addition to RANKL, osteocytes produce sclerostin, M-CSF, OPG, and other cytokines during OTM.

As the early findings in OTM, osteocyte apoptosis peaks at 24 h on the compression side in mouse OTM models, as measured by TUNEL and caspase-3 immunofluorescence stain [73]. Osteoclastogenesis was evident after 72 h and continued to increase up to 7 days. Apoptotic osteocytes were preferentially located close to osteoclasts, suggesting that dying osteocytes produce active signaling to recruit osteoclasts [73,74,75].

Osteocytes can be also an important source of RANKL in OTM mouse models [22,76,77]. Osteocyte-deleted mice have a 60% reduction in osteoclasts and a 50% reduction in tooth movement compared to normal controls [78]. Under basal conditions osteocyte ablation negatively affects bone quality by increasing intracortical porosity, osteoblastic dysfunction, and adipose tissue proliferation in the marrow space [79]. These mice showed a severe osteopetrotic phenotype due to a lack of osteoclasts. The mice with lineage-specific RANKL deletion in osteocytes decreased OTM by 40% and osteoclast number by 60% compared with WT mice [22]. In vitro, osteocytes express a higher amount of RANKL and have a greater capacity to support osteoclastogenesis than osteoblasts and bone marrow stromal cells [77]. Interestingly, the osteoblast number on the tension side was significantly reduced in the same transgenic mice, possibly through a coupling mechanism.

#### 2.2.3. Osteoblasts

Osteoblasts are bone forming cells, accounting for the 4–6% of total bone cells and differentiate from mesenchymal stem cells [80]. Runx2 and osterix are transcription factors that promote osteoblastic differentiation from mesenchymal stem cells. The fate of osteoblasts includes: (1) apoptosis, (2) become bone-lining cells or (3) form osteocytes. Bone-lining cells maintain homeostasis of bone and contain osteoblast progenitors [81]. Osteoblasts are mechanosensory cells and convert the mechanical signals into biological responses, producing various cytokines such as prostaglandin, OPG, RANKL and BMPs [82,83]. In OTM, bone-lining cells and osteoblasts express M-CSF and RANKL and produce other factors that positively influence osteoclastogenesis, including IL- 1β, IL-6 and TNF-α [84,85,86].

Osteoblast differentiation is an important process on the tension side during OTM. The initial response to OTM on the tension side is a proliferation of osteoblast progenitors that express α-SMA, which peaks at 2 days after initiating OTM while osteoid formation in mice peak at 4 days, the early phase of OTM, represented by osteopontin, osteocalcin, and bone sialoprotein in mouse OTM models [87,88]. Endothelin B receptors (ET_B_) play an important role in alveolar bone modeling in the late stage of OTM in the rat animal model [89]. To examine the role of osteoblasts in OTM, ET_B_ knockout rats (ET_B_-KO) exhibited decreased OTM after 35 days, a late stage in OTM, by 27% compared to the ET_B_-WT mice. The alveolar bone volume in the ET_B_-KO appliance group was significantly less due to diminished osteoblast activity, but osteoclast volume was not significantly different compared to the ET_B_-WT appliance group. In addition, the expression levels of osteocalcin and DMP1, the osteoblast activity markers, were significantly down-regulated by 70% in the ET_B_-KO appliance group compared to the ET_B_-WT appliance group. However, the expression of cathepsin K, an osteoclast activity marker, did not show any statistical difference. In summary, ET_B_ knockout rats (ET_B_-KO) have significantly lower osteoblast activity, unbalanced bone resorption/new bone formation, and reduced OTM with increased tooth mobility compared with control group, explaining the role of osteoblasts in the late stage of OTM.

Taken together, identifying the roles of each cells during OTM is critical and use of the transgenic mouse with each cell type-specific gene deletion can be a great tool for these studies.

### 2.3. Intracellular Signaling Pathways Stimulated by Mechanical Force

In OTM, various signaling pathways are activated, which mediate the response of mechanosensory cells that modulate bone resorption and formation. The function of Wnt/β-Catenin signaling, and Yes-associated protein and transcriptional coactivator with PDZ-binding motif (YAP/TAZ) signaling participate in bone remodeling in tooth movement.

Wnt/β-catenin signaling is critical for bone homeostasis [49,50]. β-catenin is a transcription factor that is activated by canonical Wnt signaling and translocates to the nucleus in osteoblasts lineage cells subjected to mechanical stimulation [90]. During OTM, Wnt/β-catenin signaling modulate expression of osteogenesis- and osteoclastogenesis-related factors in response to mechanotransduction [91,92,93]. Mice with global loss-of-function Lrp5, a Wnt receptor, have low bone mineral density and impaired osteogenic response to mechanical loading [94]. Conversely, mice with gain-of function mutations in the Lrp5 gene have significantly increase bone mineral density and bone mass in response to mechanical forces [95,96]. In OTM, a gain-of-function mutation in Lrp5 decreases orthodontic tooth movement by reducing osteoclast- mediated bone resorption and increasing alveolar bone mass [97]. Consistent with this, constitutive Wnt signaling increases osteogenic gene expression and reduces RANKL expression and osteoclast activity [98]. Conversely, viral transduction of DKK1, a Wnt inhibitor, increases osteoclast activity and reduces osteogenic markers, resulting in increased PDL width [98]. In a rat OTM model, the expression of Wnt3a, Wnt10b, and β-catenin is stronger on the tension side, consistent with Wnt induced bone formation observed under tension. In contrast, Dkk-1 levels are much higher on the compression side, consistent with reduced Wnt signaling and greater bone resorption on the compression side [99].

Yes-associated protein (YAP) and transcriptional coactivator with PDZ-binding motif (TAZ) play a key role in the mechanotransduction process [63,100]. YAP senses extracellular mechanical signals and translocates into nucleus to function as the coactivator of other transcription factors [62]. During OTM, YAP/TAZ signaling is observed in osteoblasts, osteocytes, osteoclasts and PDL fibroblasts and increases proportionally with the degree of orthodontic force [62,63]. Conditional deletion of YAP in PDL mesenchymal stem cells decreases osteoclast formation by 80% on the compression side and reduces tooth movement by half. In vitro cyclic stretch stimulates proliferation of PDL fibroblasts and osteoblast differentiation via YAP activation [62,101]. YAP knockdown suppresses mechanical forced-induced osteogenesis while overexpression of YAP enhances osteogenesis in PDL fibroblasts [62].

## 3. Possible Mechanosensors

**Figure 2:**
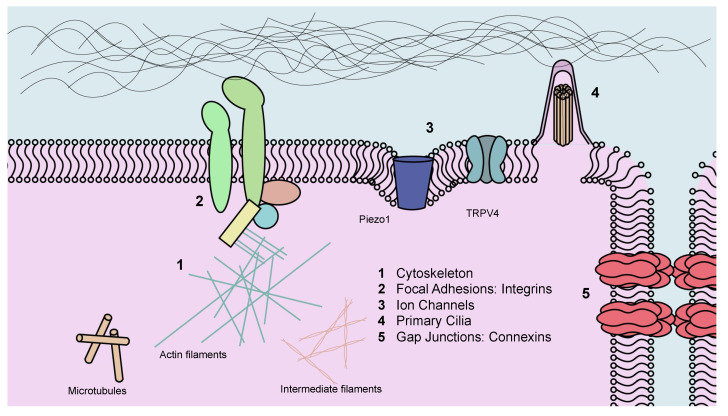
Possible Mechanosensors in Orthodontic Tooth Movement. (1) Cytoskeletons, (2) Focal adhesions: integrins, (3) Ion channels, (4) Primary cilia, and (5) Gap Junctions: connexins. TRPV4: transient receptor potential cation channel subfamily V member 4.

Cells sense their mechanical environment through cell-cell or cell-matrix adhesions during physiologic growth and development and during mechanical loading. Mechanosensing occurs by mechanical force-induced conformational changes in cellular molecules, including force-activated cytoskeleton, integrins, ion channels and cell-cell adhesions, consequently affecting cellular gene expression and its function and regulating orthodontic bone remodeling (Figure 2).

### 3.1. Cytoskeleton

Cellular cytoskeletons provide structural frameworks for the cell and are largely comprised of microtubules, actin, and intermediate filaments [102]. Cytoskeletons play a role in the response to mechanical force and are responsible for cell motility [103]. For example, cilia and flagella are mainly composed of microtubules and move as a result of microtubules sliding. In OTM, PDL and alveolar bone cells are reconstructed and their cellular cytoskeleton changes stimulate the elaboration of multiple cytokines and growth factors, mediating the cell morphology, differentiation, and proliferation [102,104,105]. On the tension side, cytoskeletal reorganization influences the differentiation of osteoprogenitors to osteoblasts and bone formation, stressing the critical role of cytoskeleton to influence both compression and tension sides during OTM [106].

### 3.2. Focal Adhesions (FAs)

Focal adhesions are integrin-associated proteins that connect intracellular actin filaments and extracellular matrix proteins [51,107]. Orthodontic force-induced stress on the extracellular matrix can be transmitted to cells through focal adhesions to induce proliferation and differentiation of several cells in the PDL and alveolar bone, leading to the balanced bone remodeling in response to the applied force [104]. Focal adhesions are involved in mechanosensing and downstream signaling through focal adhesion kinase in osteoblasts [108] and osteocytes [109,110]. Gene deletion that results in loss of focal adhesions in osteoblasts reduce mechanical responses to fluid flow [108]. Mechanical forces through focal adhesion kinases stimulate Wnt/β-catenin signaling in osteocytes [109].

### 3.3. Primary Cilia

Primary cilia are non-motile protruding organelles from the cell membrane and are observed in chondrocytes, mesenchymal stem cells, osteoblasts and osteocytes as mechanosensors [111,112]. Changes in fluid flow stimulate numerous cells via primary cilia [113], which may be important in OTM. Blocking primary cilia formation inhibits the expression of osteopontin, prostaglandins and cyclooxygenase-2 in osteoblasts or osteocytes and reduces their response to fluid flow. Tensile forces promote the osteogenic differentiation and proliferation of PDL mesenchymal stem cells via primary cilia that are needed for osteoblast differentiation and bone formation [114]. Lineage-specific deletion of key ciliary proteins including the IFT80, IFT88, Kif3a, Evc and polycystin in osteoblasts or osteoblast precursors leads to cilia loss, impairs osteoblast differentiation, reduces osteoid formation, and inhibits bone mineralization in response to mechanical loading in vivo [114,115,116]. On this basis, it may have a role in bone formation on tension side during OTM.

A calcium channel complex composed of the polycystin-1 and polycystin-2 is located at the base of primary cilium and mediates the effect of cilia bending [117]. When the primary cilium is bent by dynamic fluid flow, a Ca^2+^ signal is transduced proportional to the degree of distortion. This bending motion opens Ca^2+^-permeable ion channels and stimulates formation of inositol (1,3,5)-trisphosphate (IP3) that is transmitted through gap junctions, thereby transmitting the ciliary signal to neighboring cells [4,118,119,120]. Loss of polycystin-1 function in vivo leads to reduced formation of osteoblasts, a reduced anabolic response to mechanical loading and the development of osteopenia [121]. Conditional deletion of polycystin-1 under the control of a regulatory element of the Wnt1 promoter has been used in OTM studies [122]. Conditional polycystin-1 deletion blocks the tooth movement with reduced osteoclast formation on the compression side. This study demonstrates that the calcium channels in primary cilia play an important role in the transduction of mechanical signals to induce bone resorption.

### 3.4. Gap Junctions: Connexins

Connexins are gap junction proteins that connect two neighboring cells [123,124]. Connexin 43 (Cx43) is the most abundant connexin in bone and modulates bone resorption and formation activity by regulating osteoprotegerin and sclerostin levels [74,125]. During OTM, Cx43 is strongly expressed in osteoclasts and PDL cells on the compression side and in osteoblasts and osteocytes on the tension side in vivo [124]. In vitro studies with PDL fibroblasts report that mechanical tension increases Cx43, up-regulating the expression of Runx2 and osterix, and down-regulating RANKL expression [126]. Suppression of Cx43 reduces the induction of osteogenic markers but promotes RANKL expression [126,127]. Given its function in regulating the response of osteoblasts and osteocytes to mechanical forces, it is reasonable to speculate that connexins play a role in OTM.

### 3.5. Ion Channels

Ion channels are pore-forming membrane proteins that facilitate direct ion passage through the cell membrane [51]. Mechanical force-activated ion channels increase membrane permeability and trigger the influx of extracellular calcium, demonstrating their role in mechanotransduction in osteocytes and PDL fibroblasts [51,128,129]. Piezo1 ion channel and transient receptor potential cation channel subfamily V member 4 (TRPV4) are key factors in the mechanotransduction of osteocytes and PDL fibroblasts under mechanical loading. Conditional deletion of Piezo1 in osteoblasts and osteocytes significantly reduced bone mass and strength in mice [130]. Conversely, administration of a Piezo1 agonist to adult mice increased bone mass in a way that mimicked the effects of mechanical loading, demonstrating that Piezo1 is a mechanosensitive ion channel by which osteoblast lineage cells sense and respond to changes in mechanical load. In vitro mechanical stimulation of mature osteocytes activates Piezo1, which rapidly activates Akt and down-regulates sclerostin [131]. Piezo1 and TRPV4 increase their expression 8 h after mechanical loading, followed by the increased expression of M-CSF, RANKL and COX2 [128]. However, pretreatment with the inhibitors of Piezo1 and TRPV4 suppressed the related cytokine expression. Fluid shear stress on osteocytes activates TRPV4 to rapidly increase intracellular Ca^2+^ levels, which activates Ca^2+^/calmodulin-dependent kinase (CaMK) II and down-regulates sclerostin [132,133]. This is functionally important as shown by in vivo and in vitro studies that conditional deletion of Piezo1 in osteoblasts and osteocytes reduces bone mass and strength [130], while administration of a Piezo1 agonist increases bone mass, mimicking the effects of mechanical loading. It is likely that ion channel proteins are important in OTM.

## 4. Conclusions

Orthodontic tooth movement is a highly coordinated process in which various cells, cytokines, and complex mechanisms are involved. To date, numbers of OTM studies have been performed, but many are in vitro studies or examined the global deletion of a specific gene or cell type. Transgenic animal studies with the cell type-specific gene deletion can provide the insight into the key cellular and molecular mechanisms in OTM by establishing the cause and effect relationships. Findings from those studies could be applied for our daily orthodontic practice in the future, accelerating osteoclastogenesis and reducing treatment time. Conversely, blocking osteoclastogenesis can be applied to prevent orthodontic relapse. In addition, increasing osteogenesis can greatly help the maxillary expansion procedure, reducing the retention period of 5 to 6 months. The RANKL gene transfer to expedite the OTM is just one example. Furthermore, the findings from the transgenic animal studies can contribute to the development of precision orthodontics in the future so that we can provide patient-specific orthodontic treatment.

One of the limitations of this review is that animal studies that specifically examined mechanosensors are rare and many of them were conducted in vitro. Mechanosensors play a critical role in the mechanotransduction process and further investigation is needed. In addition, several OTM studies used slightly different amount of orthodontic force and time points. The use of standardized OTM methods would greatly help compare the outcomes from multiple animal OTM studies. Lastly, applying the findings from rodent studies to humans does warrant some modification considering the species differences, for example when considering the time periods in OTM.

## Data Availability

Not applicable.
